# Comparison of antibiotic-impregnated bone cement coverage versus vacuum sealing drainage in semi-open bone grafting using for tibial fracture with infected bone and soft tissue defect: a retrospective analysis

**DOI:** 10.1186/s12891-023-06475-x

**Published:** 2023-05-19

**Authors:** Yanlong Zhang, Shuwei Tian, Meiyu Liu, Wenfang Zhai, Yujie Zhou, Aqin Peng

**Affiliations:** 1 Trauma Center, The First Hospital of Hebei Medical University, Shijiazhuang, 050031 Hebei China; 2 Hebei Provincial General Hospital, Shijiazhuang, 050057 Hebei China; 3 The Second Hospital of Hebei Medical University, Shijiazhuang, 050051 Hebei China; 4 Department of Orthopaedics, The Third Hospital of Hebei Medical University, Shijiazhuang, 050051 Hebei China

**Keywords:** Tibial fracture, Infected bone defect, Soft tissue defect, Semiopen bone grafting, Bone cement

## Abstract

**Objective:**

To compare antibiotic-impregnated bone cement coverage (bone cement surface technique; BCS-T) versus vacuum sealing drainage (VSD) for tibial fracture with infected bone and soft tissue defect.

**Method:**

This retrospective analysis compared the clinical outcomes in patients undergoing BCS-T (n = 16) versus VSD (n = 15) for tibial fracture with infected bone and soft tissue defect at the Third Hospital of Hebei Medical University from March 2014 to August 2019. For BCS-T group, osseous cavity was filled with autograft bone graft after debridement, and then the wound was covered with a 3-mm layer of bone cement impregnated with vancomycin and gentamycin. The dressing was changed every day in the first week, and every 2 ~ 3 days in the second week. For VSD group, a negative pressure of -150 ~ -350 mmHg was maintained, and the dressing was changed every 5–7 days. All patients received antibiotics treatment based on bacterial culture results for 2 weeks.

**Results:**

The 2 groups did not differ in age, sex and key baseline characteristics, including type of Gustilo-Anderson classification, size of the bone and soft tissue defect, the percentage of primary debridement, bone transport, and the time from injury to bone grafting. The median follow-up was 18.9 months (range:12–40). The time to complete coverage of bone graft by granulation tissue was 21.2 (15.0–44.0) and 20.3 (15.0–24.0) days in the BCS-T and VSD groups, respectively (p = 0.412). The 2 groups also did not differ in wound healing time (3.3 (1.5–5.5) versus 3.2(1.5–6.5) months; p = 0.229) and bone defect healing time (5.4(3.0-9.6) versus 5.9(3.2–11.5) months; p = 0.402). However, the cost of covering material was significantly reduced in the BCS-T group (2071 ± 134 versus 5542 ± 905 yuan; p = 0.026). Paley functional classification at 12 months did not differ between the 2 groups (excellent in 87.5% versus 93.3% in the 2 groups; p = 0.306).

**Conclusion:**

BCS-T could achieve clinical outcomes similar to VSD in patients receiving bone graft for tibial fracture with infected bone and soft tissue defect, but material cost was significantly reduced. Randomized controlled trials are needed to verify our finding.

## Background

Open tibial fracture is often associated with extensive soft tissue injury and high incidence of subsequent complications, including infection and non-union [1–6]. Tibial fracture with infected bone and soft tissue defect are commonly treated with open bone grafting, fibular bone graft (either vascularized or non-vascularized), Masquelet technique, and Ilizarov bone transport, all with open wound. Open bone grafting is often conducted for bone defect < 4 cm, and involves thorough debridement before closing the wound with transfer skin flap or muscle flap [7]. Fibular graft requires expertise in microsurgery, and thus is rare outside specialized centers. The Masquelet technique involves temporary cement spacer (typically for 6–8 weeks) followed by staged bone grafting [8, 9]. Ilizarov bone transport provides axially aligned bone transportation as well as soft tissue healing support, but is painful and prolonged, and has been associated with a variety of complications due to restricted daily activities [10, 11]. Due to the advantage and disadvantage of each method, significant controversy remains in the treatment of open tibial fracture with infected bone and soft tissue defect [12, 13, 16].

A 2-stage semi-open cancellous bone grafting was first reported to treat infected small tibial bone defect with overlaying skin loss by Ueng and Shihin 1994 [14]. In the first stage, debrided osseous cavity is obliterated with synthetic bead chains impregnated with antibiotics. In the second stage, the bead chains are replaced with autologous bone graft. Wound coverage is provided by meshed porcine skin in both stages.

Starting from March 2014, we treated tibial fracture with large infected bone and soft tissue defect using an antibiotic-impregnated bone cement sheet (referred to as Bone Cement Surface Technique; BCS-T) because the porcine skin was complex to obtain, difficult to preserve, and possible transplant rejection. The current study is a retrospective analysis that included 31 consecutive cases of BCS-T treatment of tibial fracture with infected bone and soft tissue defect at our center during a period from March 2014 to August 2019. Results were compared to patients undergoing open bone grafting and vacuum sealing drainage (VSD) [6].

### Patients and methods

This study was approved by the Ethics Board of the Third Hospital of Hebei Medical University[2017-007-1]. Signed informed consent for using their data was obtained from all patients retrospectively. The inclusion criteria were as follows: (1) infected bone defects combined with soft tissue defects caused by trauma. (2) age ≥ 16 years of. (3) treated with bone cement surface technique or open bone grafting combined with VSD. (4) at least 1-year follow-up after removal of external fixator and complete clinical data.

### Open bone grafting

The wound was thoroughly debrided prior to open bone grafting with BCS-T or VSD. Removed tissue was sent for bacterial culture and antibiotic sensitivity test. For large segmental bone defect (> 6 cm), the defect was reduced with open bone transport prior to open bone grafting. For small defect in the calcaneus or tibial metaphysis, open bone grafting was performed immediately after debridement. Cancellous bone graft was obtained from the iliac crest. Antibiotic treatment was based on the results of bacterial culture, and lasted for 2 weeks.

### BCS-T group

The osseous cavity was filled with autograft cancellous bone graft after debridement, and then the wound was covered with a 3-mm layer of antibiotic-impregnated bone cement (Heraeus Medical Co., Germany). The antibiotic bone cement contains 10 gram vancomycin and 10 pieces of 80,000 unit gentamicin per 100 gram cement powder. After the cement sheet solidified, the wound was sealed with the bone cement piece and secured it with thick silk wire. The dressing was changed every day in the first week, and every 2 ~ 3 days in the second week. In most cases, dressing change was no longer needed after 2 weeks. The bone cements covering the wound were removed based on surgeon discretion (typically after 2–4 weeks). For large wound (> 3 × 4 cm), skin graft or transfer flap were considered.

### VSD group

The wound after open bone grafting was managed with VSD (Type B, Wuhan WESTIe Medical Technology, China). The pressure was maintained at -150 ~ -350 mmHg (1mmHg = 0.133Kpa) [12, 13]. Intermittent punching tube was started after 24 h. The dressing was changed every 5–7 days, and removed when the bone graft was completely covered by granulation tissue (typically after 2–4 weeks). For large wound (> 3 × 4 cm), skin graft or transfer flap were considered.

### Statistical analysis

The following measures were statistically compared between patients undergoing BCS-T versus VSD: the time to complete coverage of bone graft with granulation tissue, wound healing time and bone defect healing time, the cost of covering material, and functional leg status based on Paley classification: [17] (1) excellent: limb shortening ≤ 2 cm, malunion ≤ 7°, joint function limitation ≤ 15%; (2) good: 1 of the above 3 items not achieved; (3) poor: 2 of the above 3 items not achieved.

Continuous variables conforming to normal distribution were analyzed using Student’s t-test, and presented as mean ± standard deviation, and analyzed using Mann-Whitney U test and presented as median (interquartile range) otherwise. Categorical variables were analyzed using χ^2^ test. Statistical significance was set at p < 0.05. All statistical analyses were performed using SPSS 22.0 (SPSS Inc., Chicago, IL, USA).

## Results

The final analysis included 16 patients in the BCS-T group and 15 patients in the VSD group. The 2 groups did not differ significantly in demographics (age and sex) and key baseline characteristics, including the cause of injury, type of Gustilo-Anderson classification, [15]size of the bone and soft tissue defect, and pathogenic bacteria (Table 1). The 2 groups also did not differ in the percentage of primary debridement, bone transport, and the time from injury to bone grafting.

**Table 1:** Demographic and baseline characteristics of the patients

	BCS-T n = 16	VSD n = 15	p value
Male sex, n(%)	9(56%)	9(60%)	0.288
Age (y), mean ± SD	40.8 ± 13.1	40.3 ± 11.3	0.925
Injury cause			0.163
Traffic accident	9	9	
Bruise by heavy object	4	3	
Drifting-down	3	3	
Bone defect (cm)	3.4 ± 1.2	3.2 ± 0.9	0.499
Soft tissue defect (cm^2^)	14.2 ± 9.0	10.9 ± 4.3	0.218
G-A classification, n(%)			0.601
II	3(19%)	3(20%)	
IIIA	5(31%)	7(47%)	
IIIB	8(50%)	5(33%)	
Bacterial culture, n(%)			0.388
MRSA	9(56%)	9(60%)	
Pseudomonas aeruginosa	5(31%)	4(27%)	
Enterococcus faecalis	2(13%)	2(13%)	
Drug sensitivity			0.126
Vancomycin	7	8	
Gentamicin	6	4	
Vancomycin and Gentamicin	3	3	

**Note**: G-A classification: Gustilo-Anderson classification; **MRSA**: Methicillin-resistant staphylococcus aureus; **VSD**: Vacuum Sealing Drainage

**Fig. 1:**
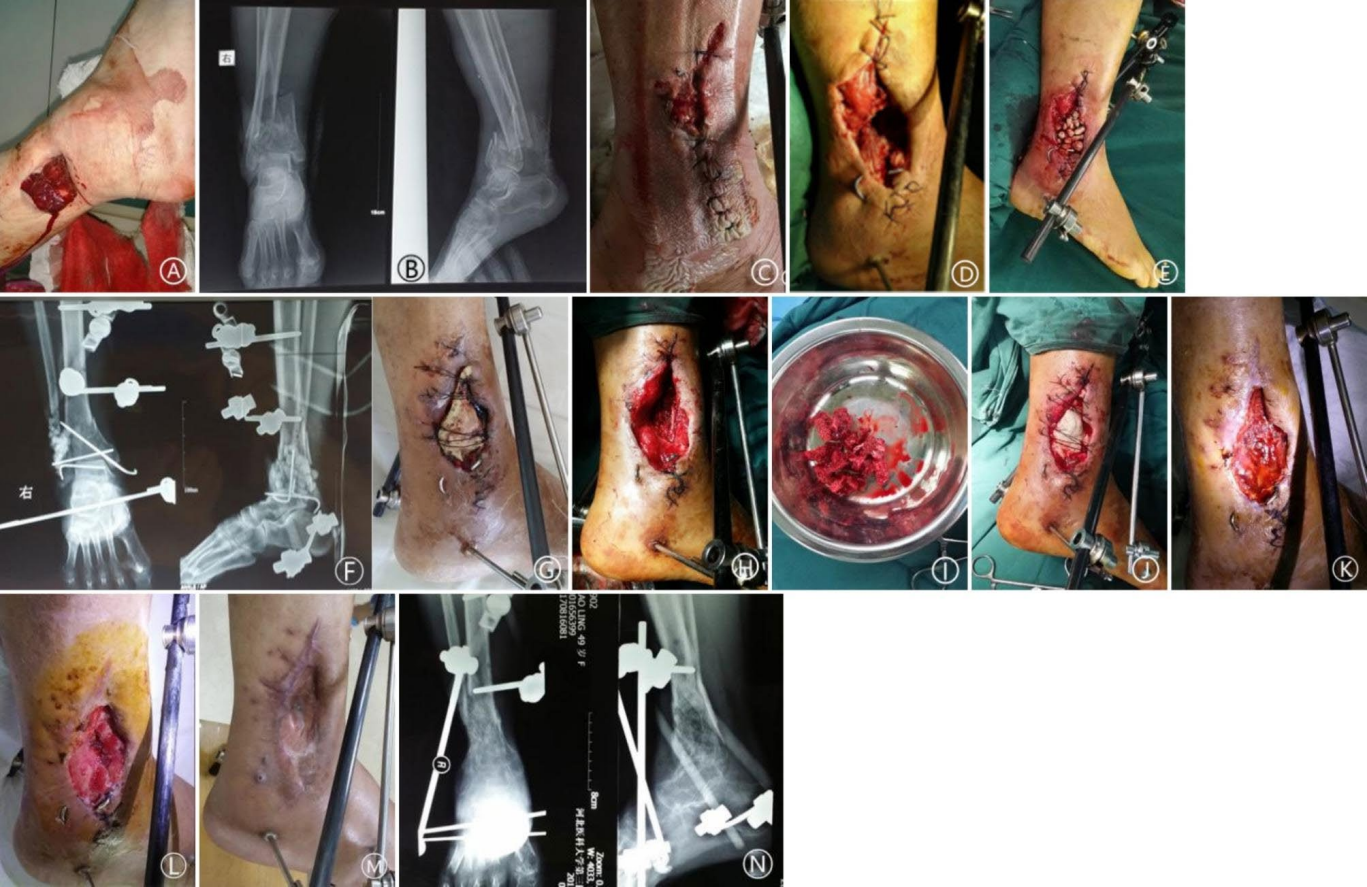
A: a 48-year-old woman presented with open fracture in the right distal tibia and 6 × 5 cm skin loss.B: X-ray. C: emergency debridement and VSD drainage. D: removal of the infected fibula and surrounding soft tissue. E: filling of the osseous cavity with antibiotic-containing cement beads. F: postoperative X-ray. G: the wound after 2 weeks of treatment. H: fresh granulation tissue upon removal of the bone cement beads. I/J: autologous bone grafting using iliac bone, followed by coverage with bone cement sheet. K/L: granulation tissue over the bone graft upon removal of the bone cement sheet 2 and 3 weeks later, respectively. M: 2 months after skin grafting. N: X-ray at 3.5 months after the bone grafting

**Fig. 2:**
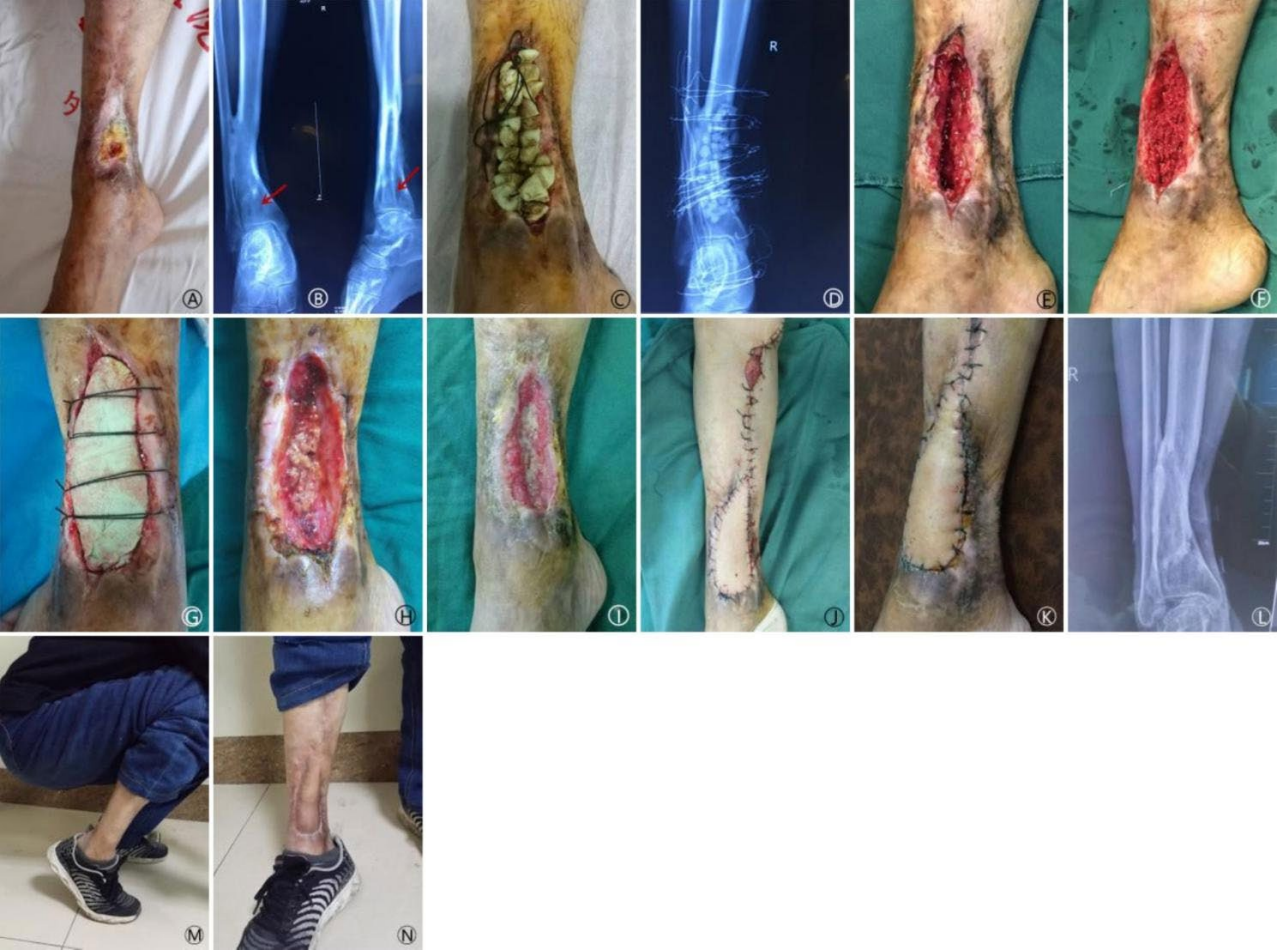
A: a 52-year-old man presented with open fracture of the right distal tibia and a sinus tract for 1 years. B: X-ray showing bone defect in the metaphysis (red arrows). C: filling of the osseous cavity with antibiotic-containing cement beads. D: X-ray at 2 weeks later. E: fresh granulation tissue. F: autologous bone grafting using iliac bone. G: coverage with bone cement sheet. H: necrotic bone graft particles upon removal of the bone cement sheet at 17 days after bone grafting. I: necrotic bone graft particles at 1 month later. J/K: medial leg flap. L: X-ray at 15 months later. M/N:ankle dorsiflexion and the wound at 15 months later

**Fig. 3:**
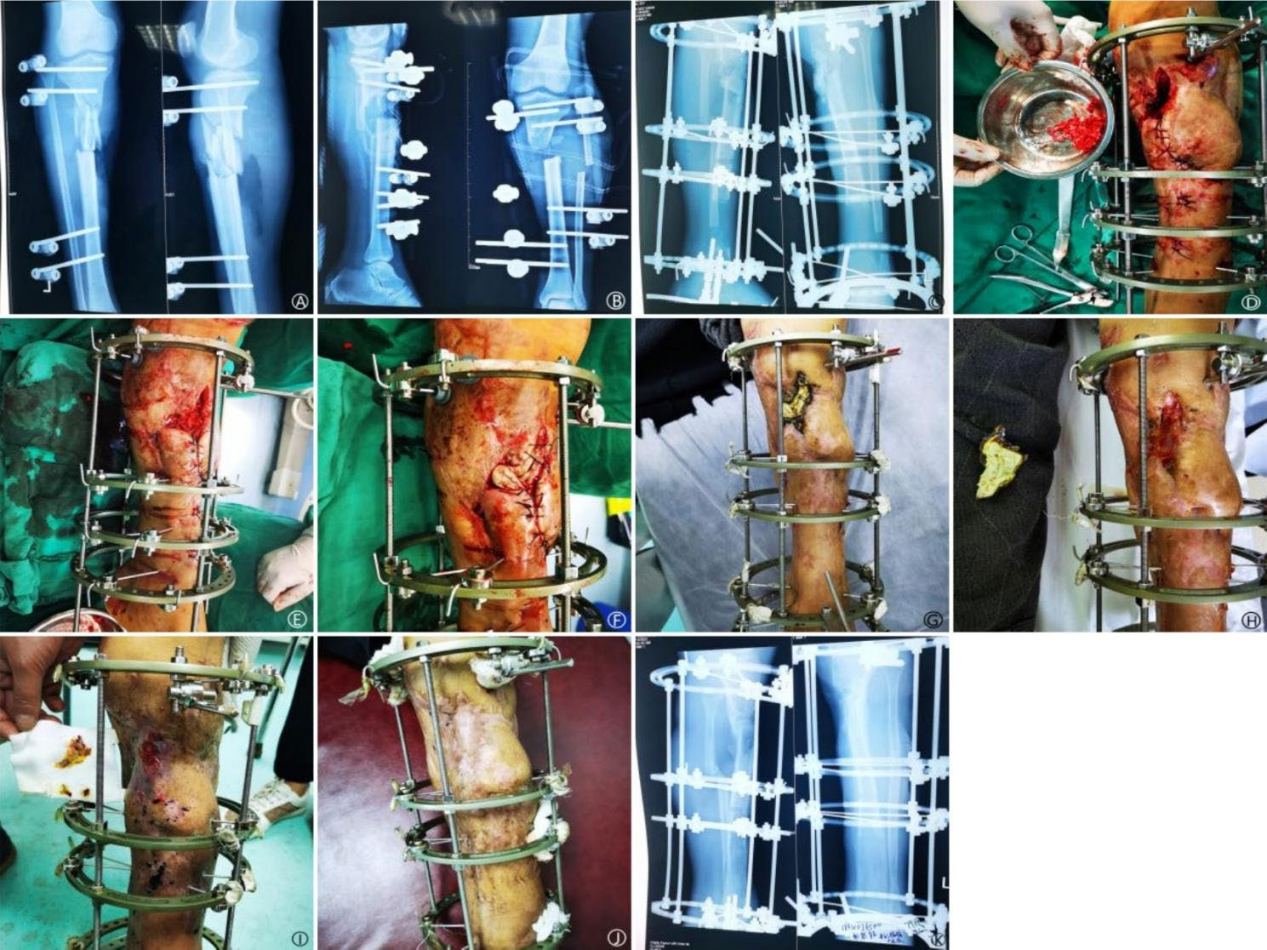
A: a 32-year-old man underwent open fracture of the left proximal tibia and wound infection after external fixation. B: after two debridements, the bone defect was 8 cm, with 2.5-cm distance between the amputated ends despite of shortening by 5.5 cm. C: X-ray at 2 months semi-open bone grafting and biplanar osteotomy. The proximal bone segment was moved 0.5 cm reversely to compress the bone graft end and the distal segment was lengthened by 6 cm to restore the length of the leg. D/E: preparation and implantation of cancellous bone graft granules from iliac bone mixed with 0.5-g vancomycin. F/G: coverage by bone cement sheet. H: fresh granulation tissue covered the bone graft at 2 months later. I/J: 4 and 6 months after bone grafting, respectively. K: X-ray at 7 months later

The average follow-up was 18.9 months (range: 12 to 40 months). The time to complete coverage of bone graft with granulation tissue was 21.2(15.0 ~ 44.0) days in the BCS-T group versus 20.3(15.0 ~ 24.0) days in the VSD group (p = 0.412) (Table 2). Among the 16 patients in the BCS-T group, the bone graft was completely covered by granulation tissue in 14 (87.5%) cases upon removing the bone cement sheet. A small amount of necrotic tissue was observed in 2 remaining cases; after secondary debridement, the bone graft was completely covered by granulation tissue in 2 weeks in one cases, and the second cases required additional bone grafting, which was completely covered by granulation tissue in 3 weeks. Among the 15 patients in the VSD group, the bone graft was completely covered by granulation tissue upon removing the VSD in 13 (86.7%) cases. A small amount of necrotic tissue was observed in 2 patients. The wound healed spontaneously after routinely dressing changes in 12 cases, and after skin grafting in the remaining 3 cases. The 2 groups did not differ in wound healing time (3.3(1.5 ~ 5.5) months in the BCS-T group versus 3.2(1.5 ~ 6.5) months in the VSD group; p = 0.229) and bone defect healing time (5.4(3.0 ~ 9.6) months in the BCS-T group versus 5.9(3.2 ~ 11.5) months in the VSD group; p = 0.402), as well as Paley classification. But the covering material cost was significantly reduced ((2071 ± 134) yuan in the BCS-T group versus (5542 ± 905) yuan in the VSD group; p = 0.026) (Table 2). Representative cases are shown in Figs. 1, 2 and 3.

**Table 2:** Clinical outcomes

Outcomes	BCS-T n = 16	VSD n = 15	P-value
T1(day)	21.2(15.0 ~ 44.0)	20.3(15.0 ~ 24.0)	0.412
T2(month)	3.3(1.5 ~ 5.5)	3.2(1.5 ~ 6.5)	0.229
T3(month)	5.4(3.0 ~ 9.6)	5.9(3.2 ~ 11.5)	0.402
Necrosis, n(%)	2(12.5%)	2(13.3%)	0.105
Paley score, n(%)			0.306
Excellent	14(87.5%)	14(93.3%)	
Good	2(12.5%)	1(6.7%)	
Cost of material (yuan)	2071 ± 134	5542 ± 905	0.026

**Note: T1**: Time of granulation tissue covering bone graft granules; **T2**: Wound healing time; **T3**: Bone defect healing time; **Necrosis**: Bone graft granules surface necrosis; **VSD**: Vacuum Sealing Drainage

## Discussion

Tibial fracture with infected bone and soft tissue defect are commonly treated with open bone grafting. Open cancellous bone grafting for infected bone defect was first reported by Papineau [18] in 1973. This is a 2- step procedure: completely debridement of the infection site first, followed by autologous cancellous bone grafting. In majority of the cases, bone graft is covered by granulation tissue after 2–3 weeks. Large wounds may require subsequent skin grafting. Huang et al. [19] improved the open cancellous bone grafting technique and combined the two steps into one. They treated 19 patients by performing open bone grafting immediately after debridement, and reported 6-month average time for fracture healing. This approach requires long period of hospitalization and is associated with nosocomial infection [14, 19].

In 1994, Ueng and Shih [14]reported semi-open bone grafting (using meshed porcine skin) in 13 patients with tibial fracture with bone and soft tissue defect, and reported a mean wound healing time of 3 months and average healing time of bone defect of 6.5 months. BCS-T in the current study is an alternative semi-open bone grafting. The cement sheet allows for adequate wound drainage. We observed rapid decrease in exudation within 1 week. The requirement for frequent wound dressing was reduced substantially versus open bone grafting. Previous studies showed that the antibiotics released from the cement sheet could achieve high concentration at the site of infection, and more importantly, could penetrate the biological membrane and dense bone cortex [5, 20−21]. For conventional open bone grafting, antibiotics must be given for 4–10 weeks [6, 22−23]. In the current study, satisfactory outcomes were achieved with systemic use of antibiotics for only 2 weeks, supporting the utility of BCS-T.

VSD for open bone grafting promotes wound drainage and reduce bacterial infection from ambient environment [6]. More importantly, VSD promotes blood flow to the site of infection and thus could expedite the healing process. For these reasons, VSD has been increasingly used to treat tibial fracture with infected bone and soft tissue defect. The results from the current study showed comparable outcome between patients managed with BCS-T versus VSD, including the time of complete coverage of the bone defect with granulation tissue, wound healing time and bone defect healing time. But the covering material cost was significantly reduced in the BCS-T group, compared with VSD group ( average 5542 yuan) due to average number of changes about 4.2 for VSD. Considering the procedural simplicity and reduced materials cost, these findings support the use of BCS-T as a viable alternative to VSD.

Open bone grafting has been used to treat bone defect as long as 8 cm [6, 13], but it is generally accepted that bone defect should not exceed 4.0 cm for open bone grafting [12, 22]. In the current study, the average bone defect was 3.3 cm, with the maximum at 6.0 cm. Whether such a limit could be extended remains to be studied.

Based on our experience, key technical aspects for using BCS-T in the treatment of tibial fracture with infected bone and soft tissue include: (1) Stage I bone grafting should be conducted only in cases with complete debridement of the bone defect. In cases with less-than optimal debridement, the osseous cavity should be filled with bone cement chain beads first; stage II bone grafting should be conducted after removing the bone cement. In the current study, stage I bone grafting was conducted in 10 out of the 16 cases. (2) Caution must be taken to avoid thermal damage to the activity of superficial bone graft granules due to the release of heat upon bone cement polymerization. After adjusting for the the size and shape of the wound, the bone cement sheet must be removed from the wound quickly to allow solidification. (3) The bone cement piece must be secured with suture to avoid falling off. (4) Additional bone grafting may be necessary for large bone defect from superficial necrotic bone graft granules removed.

## Conclusion

BCS-T is a viable alternative to VSD in managing tibial fracture with infected bone and soft tissue defect. It offers similar clinical outcomes with reduced nursing requirement and lower material cost. A key limitation in the current study was the retrospective design, and the presence of patient selection bias. Randomized controlled trials with large sample size are needed to verify our findings.

## Data Availability

The datasets used and/or analysed during the current study are available from the corresponding author on reasonable request.
